# A Comprehensive Discussion in Vaginal Cancer Based on Mechanisms, Treatments, Risk Factors and Prevention

**DOI:** 10.3389/fonc.2022.883805

**Published:** 2022-07-18

**Authors:** Sumit Kumar Baral, Partha Biswas, Md. Abu Kaium, Md. Aminul Islam, Dipta Dey, Md Al Saber, Tanjim Ishraq Rahaman, A. M, Talha Bin Emran, Md. Nazmul Hasan, Mi-Kyung Jeong, Ihn Han, Md. Ataur Rahman, Bonglee Kim

**Affiliations:** ^1^ Department of Microbiology, Jagannath University, Dhaka, Bangladesh; ^2^ Department of Genetic Engineering and Biotechnology, Faculty of Biological Science and Technology, Jashore University of Science and Technology (JUST), Jashore, Bangladesh; ^3^ ABEx Bio-Research Center, Dhaka, Bangladesh; ^4^ Biochemistry and Molecular Biology department, Life Science faculty, Bangabandhu Sheikh Mujibur Rahman Science and Technology University, Gopalgonj, Bangladesh; ^5^ Biotechnology, University of Pécs, Medical School, Pécs, Hungary; ^6^ Department of Biotechnology and Genetic Engineering, Faculty of Life Science, Bangabandhu Sheikh Mujibur Rahman Science and Technology University, Gopalganj, Bangladesh; ^7^ Department of Microbiology, Chittagong University, Chittagong, Bangladesh; ^8^ Department of Pharmacy, Begum Gulchemonara (BGC) Trust University Bangladesh, Chittagong, Bangladesh; ^9^ Department of Pharmacy, Faculty of Allied Health Sciences, Daffodil International University, Dhaka, Bangladesh; ^10^ Laboratory of Pharmaceutical Biotechnology and Bioinformatics, Department of Genetic Engineering and Biotechnology, Jashore University of Science and Technology, Jashore, Bangladesh; ^11^ Korean Medicine (KM) Convergence Research Division, Korea Institute of Oriental Medicine, Daejeon, South Korea; ^12^ Plasma Bioscience Research Center, Kwangwoon University, Seoul, South Korea; ^13^ Global Biotechnology & Biomedical Research Network (GBBRN), Department of Biotechnology and Genetic Engineering, Faculty of Biological Sciences, Islamic University, Kushtia, Bangladesh; ^14^ Department of Pathology, College of Korean Medicine, Kyung Hee University, Seoul, South Korea; ^15^ Korean Medicine-Based Drug Repositioning Cancer Research Center, College of Korean Medicine, Kyung Hee University, Seoul, South Korea

**Keywords:** vaginal cancer, postmenopausal, human papillomavirus (HPV) E6 and E7 proteins, synchronous or metachronous, carcinogenesis mechanisms, HPV type 16

## Abstract

Vaginal cancer is a rare and uncommon disease that is rarely discussed. Although vaginal cancer traditionally occurs in older postmenopausal women, the incidence of high-risk human papillomavirus (HPV)-induced cancers is increasing in younger women. Cervical cancer cells contain high-risk human papillomavirus (HPV) E6 and E7 proteins and inhibiting HPV gene expression leads the cells to stop proliferating and enter senescence. As E6, and E7 protein promoted the carcinogenesis mechanism, and here not only regulate the cellular degradation of P53, and pRb but also enhances the cell proliferation along with E6 protein targets the p53 for breakdown and subsequently promote the apoptotic cell death, and DNA repair inhibition, that is indispensable to the continue the lifecycle of the HPV. As a synchronous or metachronous tumor, vaginal cancer is frequently found in combination with cervical cancer. It is uncertain what causes invasive female vaginal organ cancer. HPV type 16 is the most often isolated HPV type in female vaginal organ cancers. Due to cancer’s rarity, case studies have provided the majority of etiologic findings. Many findings demonstrate that ring pessaries, chronic vaginitis, sexual behavior, birth trauma, obesity, vaginal chemical exposure, and viruses are all risk factors. Because of insufficient understanding and disease findings, we are trying to find the disease’s mechanism with the available data. We also address different risk factors, therapy at various stages, diagnosis, and management of vaginal cancer in this review.

## Introduction

Cervical, endometrial, or vulvar cancer causes primary or recurring illness in the vaginal area (1, 2). The size and location of the original tumor in the vaginal canal, histologic type and grade, lymphatic vascular penetration, regional lymph node metastasis, and previous therapy are all critical factors in determining the prognosis (3–6). Because of the tumor’s closeness to the rectum, bladder, and urethra, vaginal cancers are seldom amenable to curative organ-sparing surgery (4, 7). Vaginal cancer is commonly encountered in conjunction with cervical cancer as a synchronous or metachronous tumor (8, 9). Furthermore, vaginal cancer has been reported to develop the following hysterectomy for cervical cancer (10), and newer reports have found condylomatous lesions in vaginal cancer tissue, as well as the presence of human papillomavirus antigens and/or DNA (11, 12). These findings have led to speculation that vaginal and cervical cancers may share etiologic characteristics (13).

Vaginal cancer is an uncommon gynecological cancer, accounting for just 1-2 percent of all gynecological cancers in developed countries (14, 15). Patients with canal cancer have a prognosis that is determined by their age, as well as the microscopic architecture and stage of the tumor (16). Only after the elimination of cervical, urethral, or vulvar origins should cases be categorized as vaginal carcinomas, according to the International Federation of Gynecology and Obstetrics (FIGO) (15). Because stage III studies have not been conducted because of the rarity of vaginal cancer. Based on retrospective or comparative research, current guidelines have been developed. This circumstance justifies the wide range of treatments available to women who have been impacted by those who suffer from this condition are subjected to it (17).

The genital organ is made up of the internal genitalia of a woman. Eighty-five percent to ninety percent of invasive female vaginal carcinomas are epithelial cell carcinomas. Glioma, basal cell malignant neoplastic illness, sarcoma, skin cancer, and undifferentiated malignant neoplastic disease are the remaining five hitters to fifteen (18). Primary duct carcinoma accounts for around 3% of all cancers of the female reproductive system. Squamous cell carcinoma *in situ* or invasive cancer of the vaginal canal affects around 1 in 100,000 women. Only two population-based case-control studies have been undertaken to establish the etiology of this disease due to its rarity (13). In the United States, about 3,000 cases are identified each year, with approximately 900 fatalities. Ductal carcinomas are becoming more common in young women, owing to precarious HPV infection (19, 20). The channel is a frequent website of pathologic process medicine cancer and its pathologic process or direct extension of non-gynecologic tumors to the channel also can occur from the bladder, urethra, rectum, and barely the breast, lung, or alternative sites (21, 22). A 35% risk of secondary malignancies spreading to other organs, including large intestine, anal, bladder, vulvar, and skin cancers, was discovered in one study of a woman who had girdle radiation. According to the findings of several research studies, Collins and Barclay identified seven vaginal malignancies during pregnancy that were accessible for extensive examination in 1963 (23). Likewise, additional five cases have also been reported in several research studies (24–28). Because vaginal squamous cell carcinoma in pregnancy is such an uncommon occurrence, discussing survival and prognosis is challenging. Only four individuals with vaginal squamous cell carcinoma during pregnancy survived, according to the literature. Eight of the patients showed a quick progression of symptoms. Three of the four survivors reacted well to an interstitial radium needle, while the other received selective pelvic lymph node dissection followed by concomitant cisplatin chemoradiation. Only three patients had a healthy baby as a result of their pregnancy (23).

Between 1968 and 1974, no cases of duct cancer were recorded in persons who were followed up on (29). After menopause or giving delivery, the majority of women with vaginal cancer report vaginal bleeding. Patients with prevaginal cancer are more prone to experience bladder discomfort and frequent contractions. Pelvic discomfort can also be caused by infections that extend outside the vaginal canal (20). The study gives an overview of epidemiological data on vulvar and vaginal malignancies in Germany. Morbidity, prevalence, therapeutic management, and survival data are seldom consistent. The New England Journal of Public Health published the study. The most frequent precancerous activity of the vulva is neoplasia in the epithelium. After doubling in the 1980s, this rate has been steadily growing since the mid-1970s (30). This rise can be attributed to several factors, including early intercourse and promiscuity. Squamous cell carcinoma (excluding 1.1% of sarcomas) accounts for 88% of vulvar malignancies, whereas melanoma accounts for 7.5%. The remaining 4.5% is made up of adenocarcinomas, multiple entities, and other malignant tumors (31, 32). Adenocarcinomas differ from squamous disease in that they commonly metastasis to the lung, supraclavicular, and pelvic nodes, and have a peak incidence between 17 and 21 years of age. Clear cell adenocarcinomas are uncommon and mostly affect people under the age of 30. They are often linked with adenosis. In women who have been exposed to DES, adenosis is the most prevalent histologic abnormality (33). Squamous cell vaginal carcinomas spread superficially at first, but many women develop metastatic illness as a result. The lungs and the liver are common sites of distant metastases (10). Squamous cell carcinoma is diagnosed at a rate of 1.35 to 1.5 per 100,000 people in the United States. Germany’s Robert Koch Institute (RKI) and the Registry of Cancer Epidemiology approximated data from 2004 reveal a new gynecological cancer (31, 34). The incidence of cancer of the female genitals will increase with age and the height incidence is seasoned between sixty and seventy-four years more matured (31). The remaining 5% to 15% are glioma, basal cell malignant neoplastic illness, sarcoma, malignant melanoma, and dedifferentiated malignant neoplastic disease. In 2011, 4340 women in the United States were diagnosed with invasive female genital organ cancer (18). The underlying cause of invasive female genital organ cancer is unknown. In female vaginal organ malignancies, HPV type 16 is the most isolated HPV type. Women under the age of 40 are more likely to be diagnosed with breast cancer (18, 35, 36). This is why most patients are over 60 years old (16). Although there is evidence that squamous cell carcinoma rates rise with age and are greater in blacks than whites (37, 38), few additional risk factors have been found. Because of the disease’s rarity, the majority of etiologic insights have come from case studies. These studies suggest that vaginal damage from ring pessaries, chronic vaginitis, sexual behavior, birthing trauma, obesity, exposure to chemicals in the vagina, and viruses are all risk factors (13).

The works on this topic are insufficient. There is no study where we found the mechanisms, therapy, risk factors, and so on all at once. We try to focus on the processes, kinds, risk factors, therapy at various stages, diagnosis, and management of vaginal cancer in this paper.

## Diversifications of Vaginal Cancer

Generally, in the human body, there are two primary types of vaginal cancer. We have described these types in the following.

### Squamous Cell Carcinoma

According to the research published in Annals of Internal Medicine, the age-adjusted incidence of invasive squamous cell carcinoma in black women was 72% higher in white women and 34% lower in Asian/Pacific Islander women. The most frequent histological malignancy in women was squamous cell cancer, which accounted for 71% of *in situ* and 66% of invasive cases. *In situ* cancer rates peaked around 70 years old and then began to fall, although they stayed steady or rose as people became older (39).

The most prevalent kind of vaginal cancer is squamous cell carcinoma. The thin, flat epithelial cells that line the vaginal surface give rise to these malignancies (40, 41). Squamous cell carcinomas account for around 80–90% of all primary vaginal malignancies (21, 42). Squamous cell carcinoma can be classified as differentiated [G1], moderately differentiated [G2], or poorly differentiated or undifferentiated [G3] on the basis of histological findings (43). They have a sluggish growth rate and may be caused by a precancerous disease called vaginal intraepithelial neoplasia (VAIN) (10).

Vaginal intraepithelial neoplasia [VAIN] can be a precursor to squamous cell carcinoma of the vaginal mucosa, although its real malignant potential is uncertain (21, 39, 44, 45). VAIN has been found in women who have had previous gynecological cancer radiation (46). When compared to VAIN that is not linked with radiation, this kind of VAIN appears to be more prone to return following surgical excision or ablation, and to develop to invasive malignancy (39).

### Adenocarcinoma

Adenocarcinoma is the most common kind of vaginal cancer, accounting for around 15% of all cases (47). These malignancies start in the vaginal gland cells. They are more common in women over 50, but they can also happen in women whose mothers were exposed to diethylstilbestrol (DES) during pregnancy (48, 49). In 1971, a link was discovered between *in-utero* exposure to diethylstilbestrol during pregnancy and an increased risk of clear cell adenocarcinoma of the vaginal mucosa in young women. The typical age at diagnosis is 19 years, and 90% of patients are diagnosed with stage I–II illness. Neither the use of contraception nor pregnancy is linked to an increased risk of this malignancy. Endometriosis is the most common cause of vaginal endometrioid radiation (50, 51).

**Figure 1:**
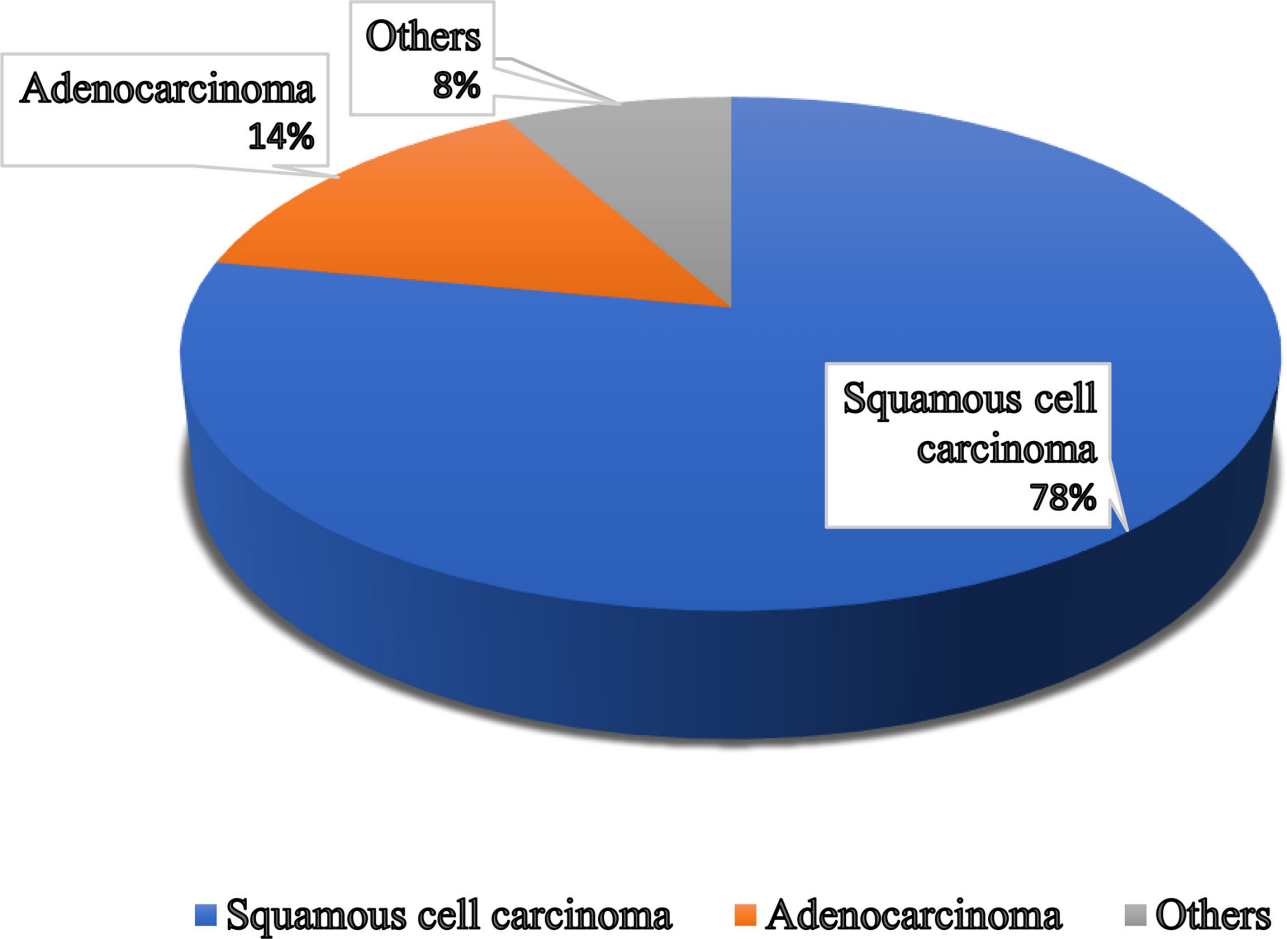
Different types of vaginal cancer and their infection rate. Various types of vaginal cancer are found in different parts of the vagina. Among them, squamous cell carcinoma, which is linked to the vaginal surface area and arises from epithelial cells, is the most prominent type of vaginal cancer. Adenocarcinoma is also found as a common type of vaginal cancer, which is responsible for 15% of all vaginal cancers. Besides, endrometriosis, testicular and some other types of cancer are noticed in the vaginal area.

Some types of cancer, such as endometriosis and testicular cancer, can develop in the vagina. Certain cancers may spread to the vagina from their original site - such as uterine, cervical, rectal, or bladder cancers (**Figure 1**). These cancers are treated according to the primary cancer type (52, 53).

## Overview of Vaginal Cancer Development Stage

**Figure 2:**
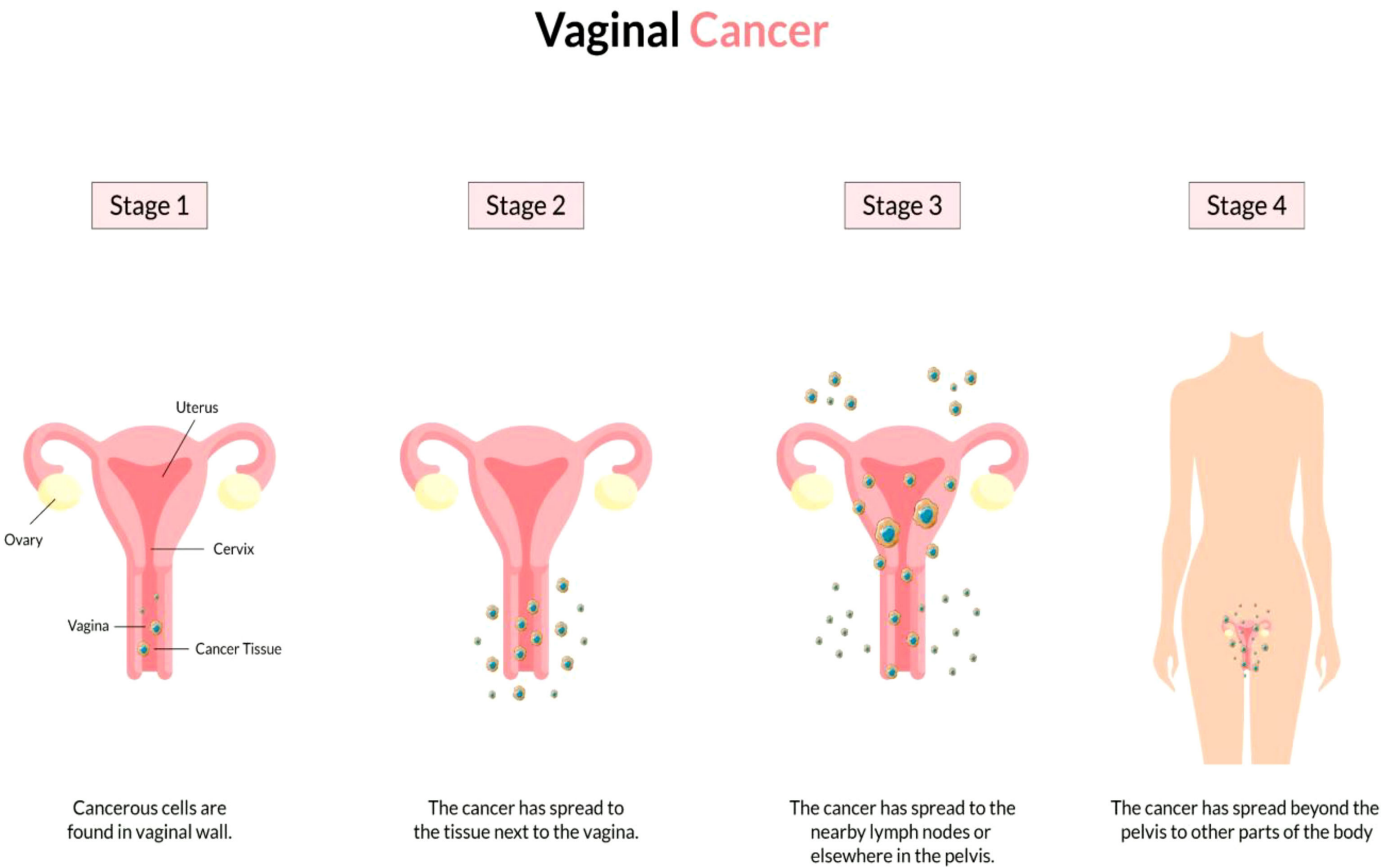
Overview of vaginal cancer and stages. Vaginal cancer is a rare kind of cancer that affects the vaginal area. The cells that line the vaginal surface are the most often affected by vaginal cancer. When found early on, vulvar cancer is generally treatable. Treatment for vaginal cancer that has progressed beyond the vagina is significantly more challenging. Stage I- Only the vulva or perineum (area between the anus and vulva) have cancerous cells. Stage II- Cancer has progressed to the urethra, anus, or vaginal region. Stage III- The malignancy has progressed to the lymph nodes in the surrounding area. Stage IV- Cancer has spread to other regions of the body from the lymph nodes.

The vagina is a 3- to a 4-inch fibromuscular tube that runs from the lower side of the cervix to the vulva. It is useful for determining the location of tumors and lymphatic drainage (54). Lymph nodes are frequently involved in vaginal cancer, even in the early stages (55). The FIGO and the American Joint Committee on Cancer have developed recommendations for staging and classifying vaginal cancer (**Figure 2**) (56). Clinical parameters are used to determine the stage of vaginal cancer. Stage I cancer is restricted to the vaginal wall; Stage II cancer has spread to the sub-vaginal tissue but not to the pelvic wall; Stage III cancer has spread to the pelvic wall, and Stage IV cancer has spread beyond the true pelvis or has reached the bladder or rectum mucosa (15, 20).

## Molecular Mechanisms of E6 and E7 Overexpression Due to Integration of Human Papilloma Virus in Vaginal cancer

Vaginal cancer is a multi-stage process that is developed by the accumulation of DNA changes of host cell genes. Oncogenes undergo epigenetic and genomic alterations as a result of these variations. The tumor suppressor genes that regulate cell cycle progression, chromosomal stability, telomeres, and apoptosis. However, the key step for carcinogenesis seems to be to integrate the viral genome into the human.

Human papillomavirus (HPV) enters the vagina and causes vaginal cancer. In HPV-mediated carcinogenesis, integrating HPV DNA into the host cell genome is crucial, resulting in abnormal proliferation and evil development (57, 58). This integration of viral DNA affects the host genome by promoting oncogenes and disrupting genes, leading to inter and intra chromosome rearrangements. Two tumor suppressor genes, such as p53 and pRb, maintain the cell’s normal proliferative function. Still, the problem is that when human papillomavirus enters the cell, E6 and E7 genes play a crucial role in interfering with the tumor suppressor gene’s function (59). The interaction between E7 and pRb protein leads to its breakdown and an abnormal start of the S-phase and the E2F transcriptional factor’s discharge that stimulates cyclones and other regulatory agents (60, 61). E6 and E7 induced carcinogenesis mechanism does not only regulate the cellular degradation of two tumor suppressor genes but also enhances the cell proliferation, and simultaneously. E6 protein targets p53 for breakdown and results in apoptotic cell death as well as DNA repair inhibition, which are essential to the entire lifecycle of the HPV (62). This is crucial for vaginal cancer development because it impedes the efficiency of oxidative stress to the DNA and enables secondary mutations to accumulate.

The intermolecular interaction between E6 and E7, known as the HPV interactome, regulates the gene expression profile and intracellular signaling pathways and changes the structure of epithelial cells (63). E6 also binds to and degrades the FAS-associated death domain protein (FADD), inhibiting Fas-mediated apoptosis (64). All these molecular changes facilitate the resistance to programmed cell death. Caspases are the main molecular participants in the programmed cell death regulatory network. Therefore, the continued action of proteins E6 and E7 causes abnormal cell proliferation, mutation of the oncogene, and eventually vaginal cancer (65, 66).

**Figure 3:**
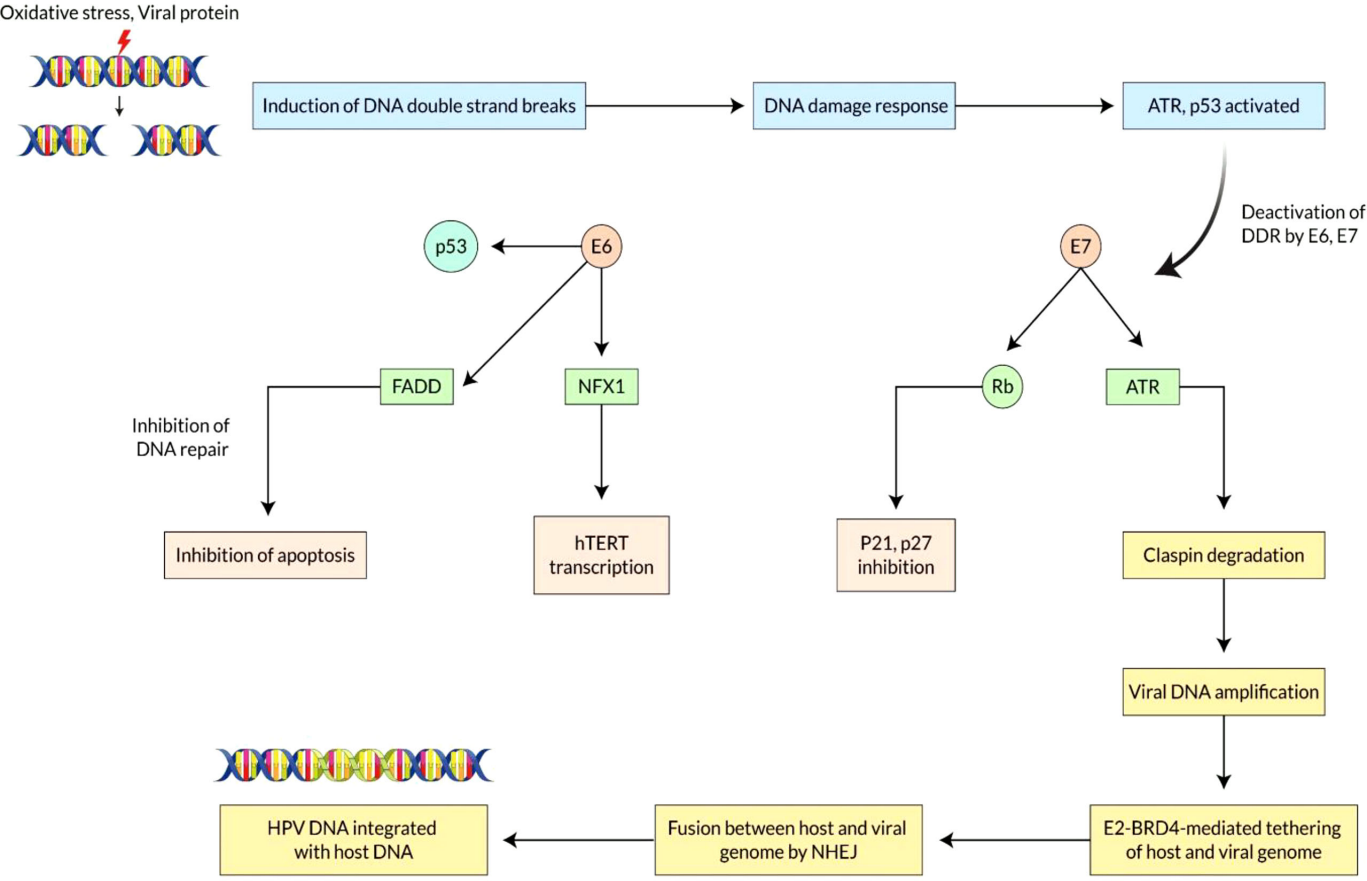
Mechanisms of E6 and E7 overexpression due to integration of Human Papilloma Virus in Vaginal cancer. Vaginal cancer is a multi-stage process that is developed by the accumulation of DNA changes of host cell genes. Integrating HPV DNA into the host cell genome is crucial, resulting in abnormal proliferation and development. E6 and E7 genes play a crucial role in interfering with the tumor suppressor gene’s function. The continued action of proteins E6 and E7 causes abnormal cell proliferation, mutation of the oncogene, and eventually vaginal cancer. The integration site is dispersed over the entire genome as chromosomally fragile areas where DNA cracks in double strands are not repaired. HPV DNA breaks are initiated when the virus is replicated, and these breaks cannot be remedied. When CDKs inhibitors (P21, P27) and P53 protein are inhibited, E6 and E7 oncoproteins damage the checkpoint of the cell cycle. DNA damaging reaction pathways regulate fusion between the two genomes by homologous or heterologous recombination. HPV genome directly binds to the host chromosome *via* HPVE2-BRD4 complex for dividing genomes into daughter cells.

Analysis of the molecular mechanism of HPV integration and overview of E6/E7 overexpression Integration usually leads to greater expression and consistency of viral oncogenic transcripts (E6 and E7) that are recognized to inactivate or speed up the degradation of diverse cellular proteins, such as the retinoblastoma protein (E7) and p53 (E6). The integration site is dispersed over the entire genome as chromosomally fragile areas where DNA cracks in double strands are not repaired (67). Integration begins with DNA strand breaks caused by oxidative stress or HPV protein, followed by DNA damage responses. HPV DNA breaks are initiated when the virus is replicated, and these breaks cannot be remedied. This injury response channel is used by HPV to generate a sufficient number of episomal HPVs, which enhance the presence of more HPV DNA for inclusion into the host DNA. At the time of DNA breaking, some DNA damage reaction (DDR) stimulates the deposition of replication factors in the replication focal area and acts as an incentive for viral replication (68). The virus uses DDR machinery to boost viral amplification, while the oncoprotein of the virus makes the cells cope with harmful downstream effects. When CDKs inhibitors (P21, P27) and P53 protein are inhibited, E6 and E7 oncoproteins damage the checkpoint of the cell cycle. The oncoprotein PV-16 E7 mitigates the reaction of the DNA damage control point by boosting up the claspin proteolytic revenue during ATR/CHK1 signaling and DNA damage control point retrieval in the cell cycle at phase G2 (**Figure 3**) (69).

Moreover, E2-BRD4-mediated therapies could enhance the viability of viral inclusion into the host genomes. DNA damaging reaction pathways such as ATM/ATR and DNA PK pathway regulate fusion between the two genomes by homologous or heterologous recombination (70). The combination of tandem arrangement in the vaginal cancer cell’s chromosome shows that a linear concentric HPV genome is produced by a rolling-cycle replication mechanism integrated into the host genome (71). It has already been revealed that only crumbled replication fork during the replication of viral genomes is created by homologous recombination machines recruited to the territories of dual strand breaks. HPV genome directly binds to the host chromosome *via* HPVE2-BRD4 (Bromodomain protein 4) complex for dividing genomes into daughter cells (72). The link between BRD4 and the fragile chromosomal area indicates that BRD4 can significantly enhance mechanistic assimilation viability. At last, the destruction of E2 ORF throughout inclusion led to increased expression of E6 and E7 viral oncogenes, leading to disruption of the critical cell genome and causing cellular death.

## Risk Factors

The most frequently detected types of HPV can be found in most vaginal tumors and HPV 16 (73, 74). Nearly 60% of the intrusive cell carcinomas and 80% to 90% of in situ, constitute HPV-DNA. The threat of *in-situ* tumors will double with HPV18. Invasive carcinoma probability is approximately six times increasing with the existence of HPV 16 antibodies. The health issues for vaginal intraepithelial neoplasia (VAIN) or vaginal cancer, particularly HPV-16 infection, were associated in a community retrospective analysis with 156 patients (75). The probability of *in situ* vaginal cancer has been reported to enhance almost six times by genital warts, but the connections with infective tumors are less clear (76). There has also been an improved risk for persons with an earlier SCC, which may be associated with HPV infection (77). About 30% of patients identified with preliminary stages have suffered before five years from invasive vaginal cancer (78). It has been reported that radiation treatment could occupy a part in the development of intermediate vaginal cancer in youth patients. There have also been higher chances for vaginal cancer for female patients with cancers in plenty of different anogenital locations or with an anogenital familial history (79). Lower educated communities and individuals living under economic conditions are associated with the risk of higher vaginal cancer (36, 80, 81).

## Updated Vaginal Cancer Identification Approaches

Bleeding, discharge, and urine retention are the most significant signs of vaginal cancer. Rectal symptoms such as constipation or blood in the stool might be caused by tumors that grow on the posterior vaginal wall. The most essential technique for determining the local extent of vaginal cancer is still a pelvic examination (15, 82, 83).

## Treatment of Vaginal Cancer Based on its Different Stages

Vaginal cancer is so uncommon as a result, there are lots of debate and disagreement over the perfect treatment. Radiation and resection can be applied in vaginal cancer if the diagnosis can be done in the early stage (**Table 1**) (84). According to FIGO, treatment should be tailored based on the stage of diseases and the location of vaginal attachment.

**Table 1:** Treatment of vaginal cancer by International Federation of Gynecology and Obstetrics’ stage.

Stage	Extent of tumor	Treatment methods
I	Confined to vagina	Small and minimum invasive tumors are considered for surgery by using EBRT with BT (84, 85).
II	Paravaginal tissue	EBRT with BT (86).
III	Pelvic wall	EBRT with brachytherapy or only EBRT (85, 87–89).
IV	rectum or bladder	EBRT with brachytherapy or only EBRT (85–87, 89).
IVB	metastasis	Palliative EBRT combination with chemotherapy (15, 90, 91).

### Stage I

**Figure 4:**
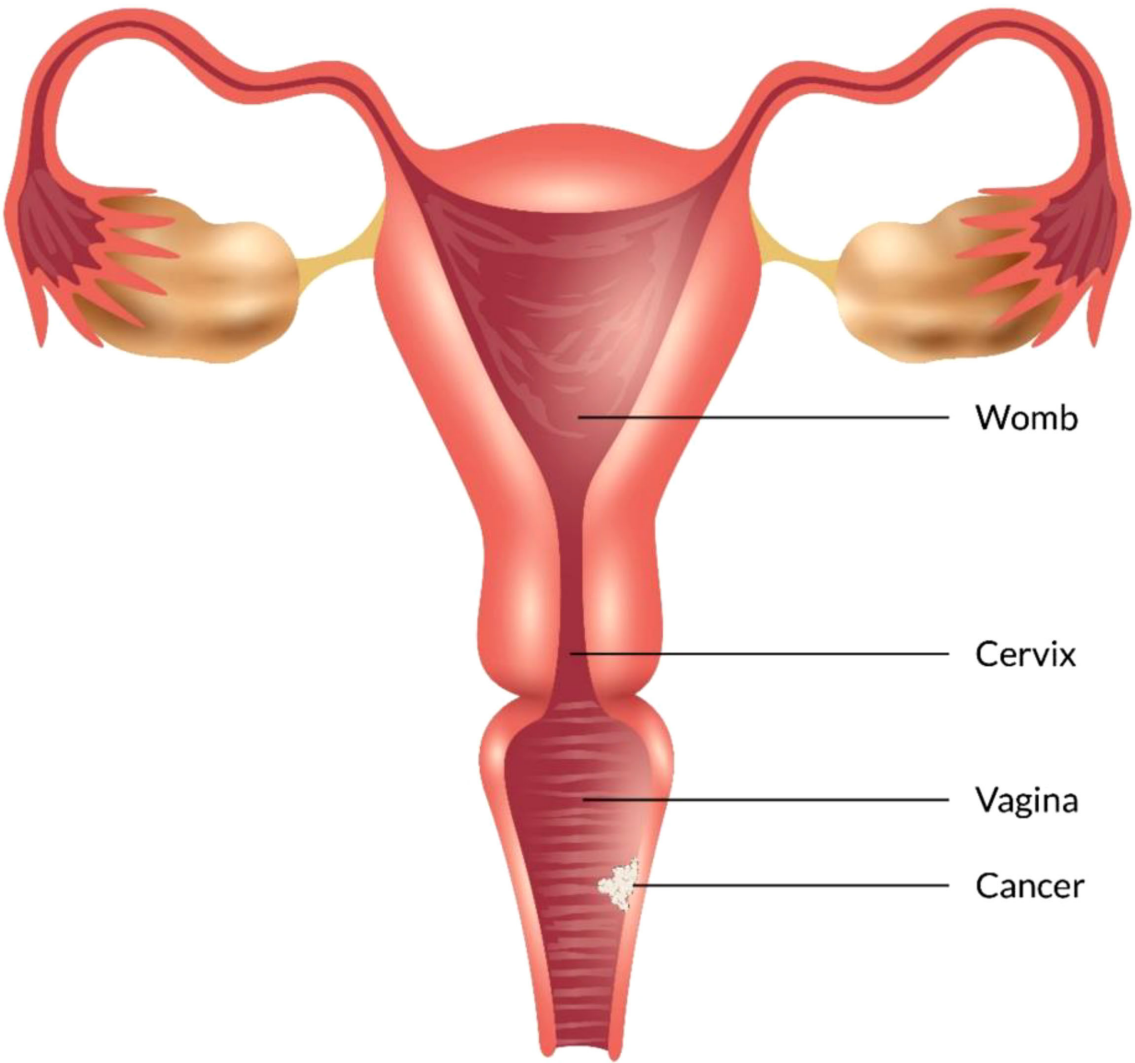
Vaginal Cancer Stage I.

In this stage, tumors are restricted to the vaginal mucosa which is classified as FIGO stage I. Radiotherapy combination with surgery or only radiotherapy is an effective treatment for FIGO stage I vaginal cancer. In FIGO stage I, the lymph nodal involvement rate is minimal which ranges from 6 to 16% (90, 92). As the surgical method is needed to be thoroughly standardized, a variety of techniques have been documented. For the selected patients who possess tiny neoplasms mass excision has been performed (**Figure 4**) (84, 92–94). There are also several adopted procedures such as entire simple vaginectomy (84, 93, 94), partial vaginectomy (92), radical vaginectomy (82, 84, 92, 93). The affected vaginal tissue is removed to the pelvic sidewall in radical vaginectomy (93). In order to perform negative margins, the lower portion of the vagina may require vulva vaginectomy (82, 92, 93). Several studies (82, 84, 92, 93, 95–97) show that 174 patients with a cumulative 5 years survival 56-90% were treated only surgery in FIGO stage I. Some writers also argued that adjuvant radiotherapy should be used for the patients who are at high risk of recurrence, with 5 years survival rate of 79-100% (55, 82, 92, 95, 96, 98–100). Combining of intracavitary and interstitial therapy (55, 82, 86, 94–96, 99, 101, 102) with EBRT in patients with increasing risk prognostic factor (6, 55, 95, 96, 99, 100, 103) was the most after used radiotherapeutic technique. The EBRT is often recommended for poor differentiated or large tumors (6, 88). In most cancer patients with advanced stages, radiation which consists of brachytherapy and bean radiation is important to treat vaginal cancer treatment (85). Radiation is beneficial because of its preservation on the vagina (85, 104). CT simulation is used for 3D conformal treatment planning which results in tumor dosage with better efficacy.

### Stage II

**Figure 5:**
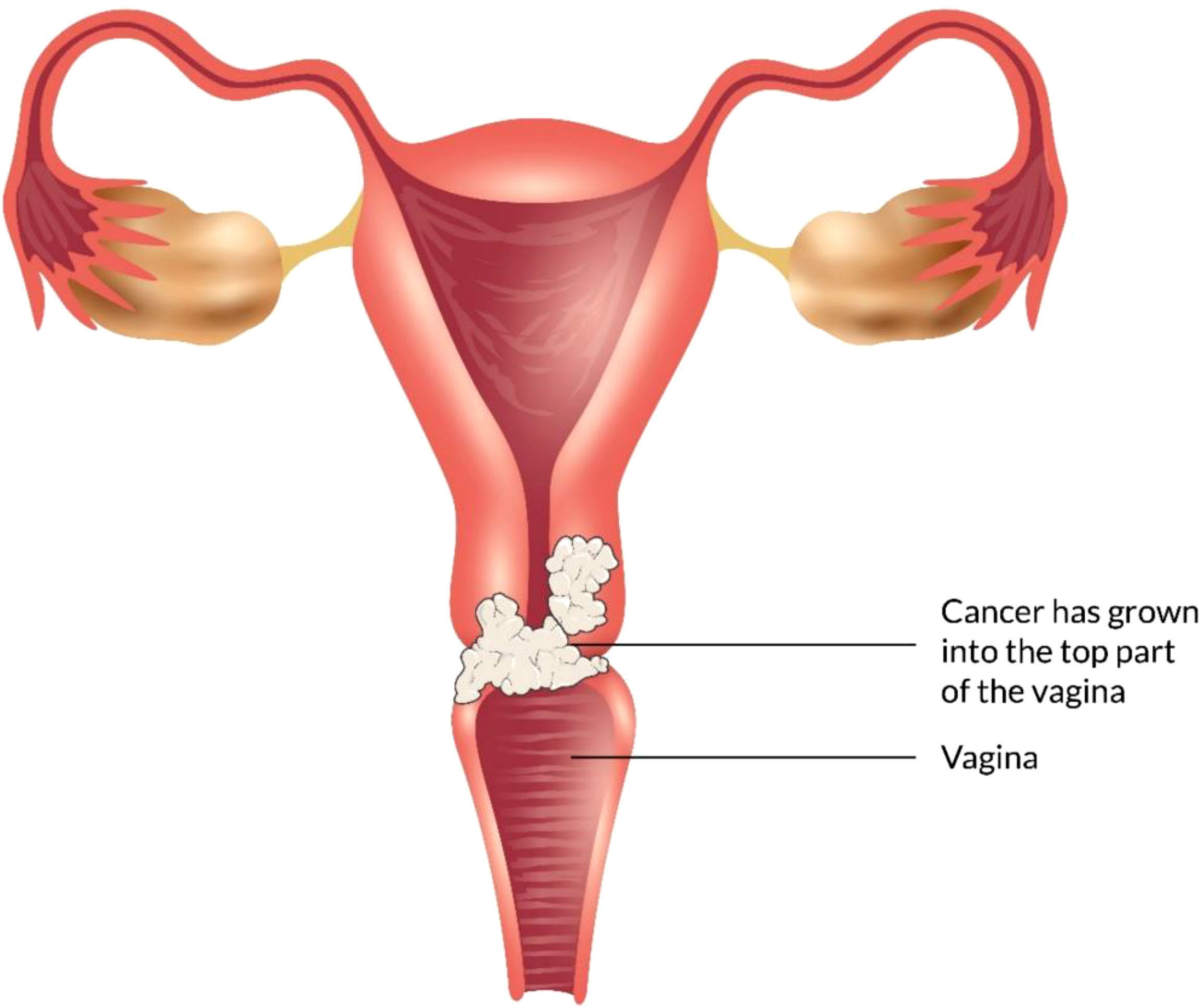
Vaginal Cancer Stage II.

In stage II vaginal cancer, the neoplasia affects the area of sub vaginal tissue but does not reach the pelvic wall (**Figure 5**). Radiotherapy is considered the most prevalent treatment method for stage II cancer disease. A mix of branchy therapy and EBRT is commonly used in standard radiation treatment (86). Only radical surgery or radical surgery combination with radiotherapy are generally preferred (55, 92). For the treatment of stage II-IVA vaginal cancer, chemotherapy is commonly recommended as a radiosensitizer. Based on randomized data Perez et al, reported that 30% of patients are in stage II and 50% are in stage III. Several strategies can be utilized to increase the radiation dosage to the main tumor location. The size and location of the tumor dictate the treatment strategy chosen. By using 5-FU and mitomycin with radiotherapy and concurrent chemotherapy, it is reported to show promising results in vaginal cancer treatment (105). To assess the therapeutic efficiency of chemotherapeutic or chemo radio therapeutic regimen, more research is needed.

### Stage III/IV

**Figure 6:**
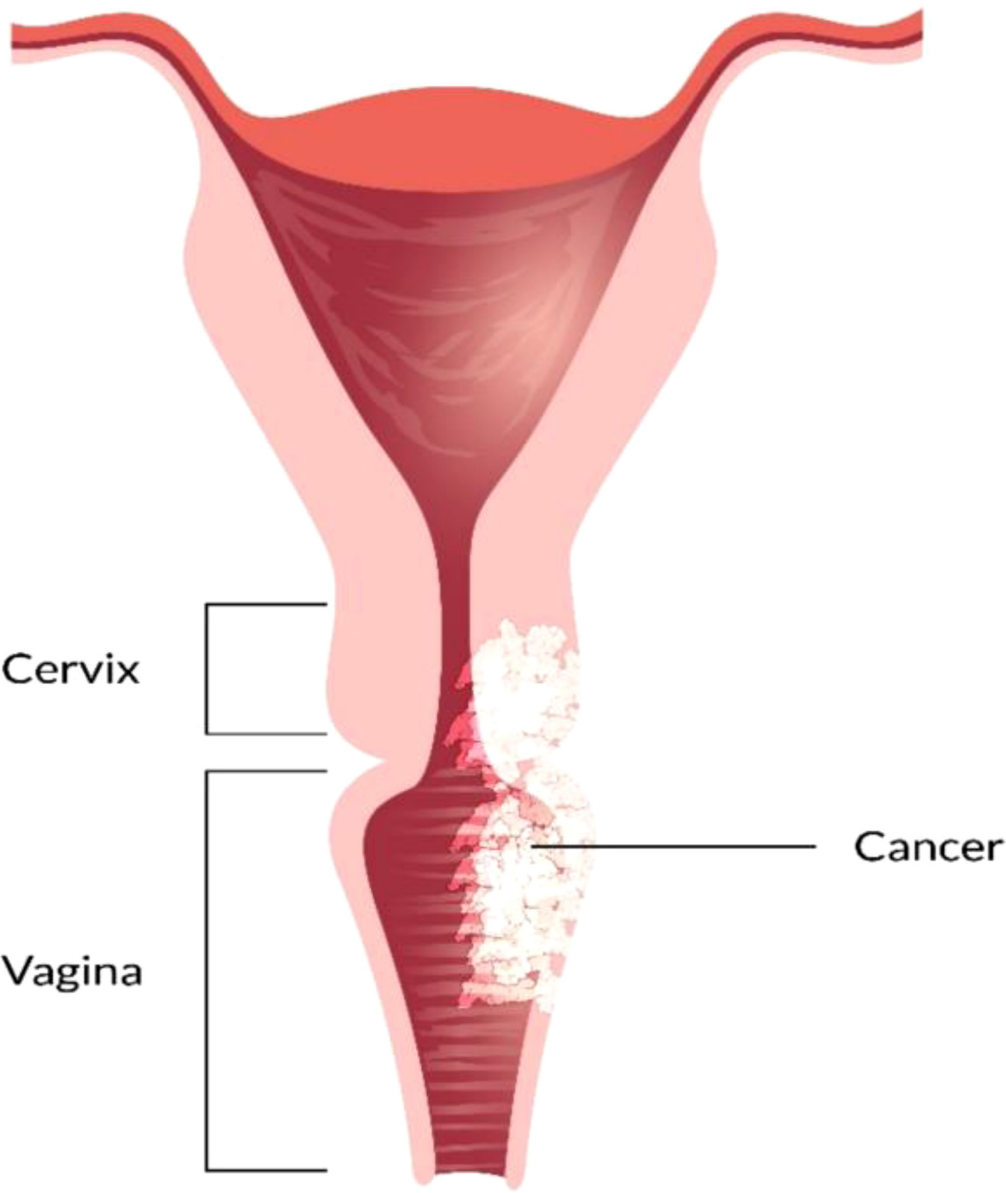
Vaginal Cancer Stage III.

**Figure 7:**
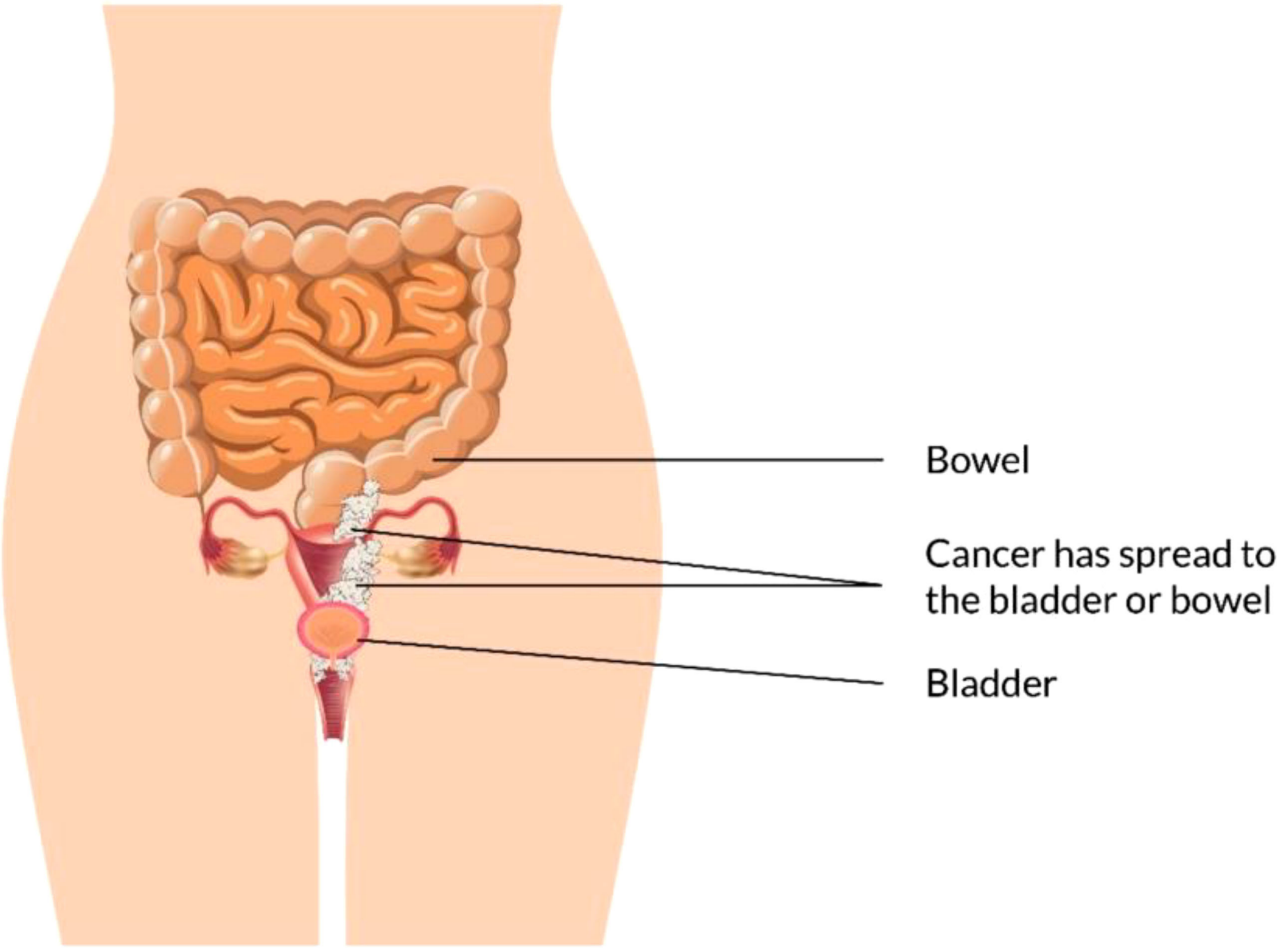
Vaginal Cancer Stage IV.

EBRT with branchy therapy or only EBRT is used for the treatment of stage III cancer disease. In females with stage III (**Figure 6**) and stage IV (advanced) (**Figure 7**) vaginal cancer, combined chemoradiation is found to perform higher metabolic and clinical response (106). EBRT combination with branchy therapy or only EBRT is used for the treatment of IVA cancer disease which is similar to the treatment of stage III cancer disease (55, 87, 89).

Palliative radiotherapy combined with chemotherapy is usually suggested for stage IVB vaginal cancer disease treatment (15, 25–27, 90, 91).

## Other Possible Strategies for The Prevention of Vaginal Cancer

The vagina is a muscular, tube-like structure that stretches from the cervix where vaginal cancer is generally developed and a very uncommon type of cancer. Vaccination against HPV (the Human Papillomavirus) giving up smoking and safer sex are all recommended by NYU Langone specialists (107).

### HPV Vaccination:

HPV has been associated with at least five other mucosal cancers: vaginal, vulvar, anal, penile, and oropharyngeal cancers. HPV is the most prevalent sexually transmitted infection in the world, with 50-80% of sexually active people developing it at some point in their life (108–110).. Vaginal cancer is responsible for 95% of all cervical cancer cases, as well as the majority of HPV-related morbidity and death. The Human Papillomavirus vaccine Gardasil has been licensed by U.S. FDA (Food and Drug Administration) to prevent precancer and cancer disease in the vagina. Gardasil also assists to prevent infections from the most common types of the strain of HPV (111, 112). Because they attack the skin, anogenital, and oral mucosal epithelial cells (108).

### Regular Gynecologic Examinations

Women who possess risk factors for vaginal cancer, need to assume early-stage gynecologic examinations regularly which will assist to prevent precancerous and cancer in the vaginal area (113). The doctor will examine the vagina, uterus, cervix and other reproductive organs also to check for any unexpected abnormalities in these parts of a women’s body (114). The Pap test, which examines the epithelial lesion to detect vaginal cancer, Cervicography, which captures a vaginal picture and analyzes the vaginal size to detect vaginal cancer, and others are among the diagnostics available for detecting vaginal cancer (115).. Various types of cancer are developed by various factors. Scientists are still looking to find out the cause for genital vaginal cancer development and solutions to prevent it (116).

## Discussion

Female vaginal cancer appears to be a malignancy that affects women in their older years of life, with the highest prevalence happening between the sixth and seventh decades of their lives. It is estimated that 15 percent of patients are diagnosed before the age of 50 and that 10 percent of tumor cells emerge in people under the age of 40 (95, 98, 117, 118). Squamous cell carcinomas account for more than 90% of vaginal cancer cases, while adenocarcinomas account for approximately 5% (95, 117, 118). Although squamous cell vaginal carcinomas immediately expanded superficially, several women were eventually diagnosed with metastatic cancer stage (10). Adenocarcinomas are distinct from squamous cancer in that they occur most frequently between the ages of 17 and 21 and often metastasize to the supraclavicular, pelvic nodes, or lung. It is rare to develop clear cell adenocarcinoma, and it usually affects people under the age of thirty. They are often linked with adenosis. Adenosis is the most prevalent histological abnormality in DES-exposed pregnant mothers (33). p53 mutations are common in HPV-negative malignancies in older women, and they are linked to an increased risk of mortality. The following are risk factors for SCC vaginal cancer: first intercourse before the age of seventeen, five or more sexual partners (83), low socioeconomic status, smoking, prior abnormal cytology, a history of genital warts, and prior hysterectomy (if the patient has had a hysterectomy) (13, 75, 119). Chronic irritating vaginitis, particularly because of constant exposure to foreign bodies like vaginal pessary and HPV infections, was associated with invasive vaginal carcinoma (120, 121). Patients who have previously developed cervical carcinoma are at increased risk of developing vaginal carcinoma, which is thought to be due to the fact that these sites are exposed to or susceptible to the same carcinogenic stimuli, whether endogenous or exogenous (82, 104).

When human papillomavirus enters the cell, E6 and E7 genes play a crucial role in interfering with the tumor suppressor gene’s function. The interaction between E7 and pRb protein leads to its breakdown and an abnormal start of the S-phase and the E2F transcriptional factor’s discharge. This is crucial for vaginal cancer development because it impedes the efficiency of oxidative stress to the DNA and enables secondary mutations to accumulate (57–59). The protein E6 regulates the gene expression profile and intracellular signaling pathways and changes the structure of epithelial cells. E6 binds and breaks down the FAS-associated death domain protein (FADD), inhibiting apoptosis-inducing communication capability through the Fas pathway. The continued action of proteins E6 and E7 causes abnormal cell proliferation, mutation of the oncogene, and eventually vaginal cancer (61, 65, 66). Viral oncogenic transcripts E6 and E7 are recognized to inactivate or speed up the degradation of diverse cellular proteins, such as retinoblastoma protein (E7) and p53 (E6). HPV DNA breaks are initiated when the virus is replicated, and these breaks cannot be remedied. The HPV genome directly binds to the host chromosome *via* the HPVE2-BRD4 (Bromodomain protein 4) complex for dividing genomes into daughter cells. BRD4 can significantly enhance the mechanistic assimilation viability of viral genomes into the host (69–71).

The stage of this cancer heavily influences prognosis. Indeed, the tumor stage strongly correlates with survival, distant metastasis, and local control (15). Additionally, the size of the tumor is used as a predictor of survival and regional or distant control (88, 122). Chyle and colleagues found that lesions with maximum diameters of less than 5 cm had a 10-year local recurrence rate of 20%. In contrast, lesions with maximum diameters greater than 5 cm had a recurrence rate of 40% (88). In particular, it is claimed in the series of Perez et al. that the lesion size is a valid predictor of pelvic tumor control and disease-free life, but only in Stage 2, when paravaginal submucosal extension is not involved with parametrial (6, 18). It has been shown that the tumor size is not a significant prognostic factor in Stages 1, II with parametrial involvement, or Stage III.

Another significant consideration while evaluating a vaginal lesion is its location. However, the effect of the position of the lesion on prognosis is controversial. According to Tarraza et al., upper-third lesions have more local recurrences, whereas lower-third lesions have a higher number of sidewall and distant recurrences (123). However, a larger series could not detect any site difference based on the location of the primary lesion (86). Many researchers have demonstrated that patients with cancers affecting the proximal half of the vagina have better overall survival and fewer recurrences than those with cancers affecting the distal half of the vagina or those affecting the entire length of the vagina, according to their findings (88, 103, 124, 125). Aside from that, lesions involving the posterior wall have a poorer prognosis than lesions involving the other vaginal walls (88, 98).

Additionally, the tumor’s location is significant as a predictor of lymph nodal status. In accordance with one theory, the efferent lymphatics from the upper section of the vaginal canal drain largely into the internal and external iliac lymph nodes, with the interiliac lymph nodes receiving the greatest amount of outflow. The lower part of the vagina lymph is drained directly into the inguino-femoral lymph nodes. Depending on the individual, other routes may also lead to the presacral or common iliac nodes (126). A higher incidence of lymph node metastasis (65%) was observed in lesions involving the posterior wall as opposed to the anterior wall or vaginal apex when the lesions were first discovered (16%) (92). The presence of nodes in vaginal cancer is thought to be a prognostic factor (6, 92, 103). Finally, the significance of age as a prognostic factor remains debatable. In some studies, the age of the patients is considered a prognostic factor; however, in other studies, this is not the case (6, 98, 125, 127). In terms of histologic type and grade, several studies have demonstrated that grading is a reliable and significant predictor of survival in the absence of other risk factors (103, 122, 125). Additionally, Chyle et al. (88) demonstrated that individuals with adenocarcinoma experienced a significantly higher rate of local recurrence than patients with squamous cell carcinoma.

In most situations, the prognosis is given too late, by which time the disease has spread to the submucosal tissue and, sometimes in cases, the pelvic sidewall of the patient, resulting in death. Even more challenging, the syndrome is most frequent in older persons, making treatment even more difficult to obtain. At the moment, radiation is used to treat the great majority of malignant tumors. Brachytherapy can be used on the primary tumor, whereas external beam radiation can be used on metastasized lymph nodes.

In the initial stages of vulvar, only surgery is considered as the backbone of treatment, whereas in advanced stages, surgery is combined with chemotherapy or radiation (128–130). Surgical intervention ensures adequate local control, but it comes with anatomical or functional limitations that compromise quality of life and sexual function. In recent times, a more comprehensive surgical treatment has been performed for the selected persons which permit anatomic reconstruction (131–133). By using tension-free skin closure, reconstructive surgery should be able to replace the lost tissue with a healthy donor region. Despite the fact that several reconstructive surgical treatments have been offered throughout the years, the prevalence of postoperative problems continues to be significant (134, 135). Furthermore, a significant complication rate affects surgical consequences for malignant gynecological pathologist (136). Wound dehiscences, low genital tract infections, vaginal and anovaginal shrinkage, urethral damage, and anti-aesthetic reconstructions, in particular, might exacerbate the patient’s quality of life and necessitate reintervention (137). The fasciocutaneous advancement flaps are the most commonly utilized by oncologists and gynecologists because of their ease of usage and good surgical results (137). With V-Y fasciocutaneous flap repair, Hand et al. found 33 and 15% surgical dehiscence and infection, respectively (138). The vitality of the restored tissue following its displacement is an important factor in vulvar flap success (128). Some points about reconstructive surgery. V-Y advancement gluteal fold flap (VYGF), Medial thigh fascio-cutaneous flap (MTF), Lotus petal flap (LPF) etc (131). The fascio-cutaneous flasps are distinguished from other type’s flasps by their simple feasibility, lower donor site morbidity and thus, they are frequently performed by oncologist (139). The operation has traditionally been reserved for the very beginning stages of this cancer. Improved anesthesia has made it possible to subject a larger number of patients to surgery, including those who are elderly or have comorbidities, and the discovery that tumors of the lower genital tract are chemo-sensitive, as demonstrated by several neoadjuvant chemotherapy trials, the field of urology has seen a resurgence in recent years (140–142). As time progresses, it is expected that surgery will play an increasingly important role. When comparing surgery to radiotherapy alone in stage I, investigations conducted on retrospective series have consistently demonstrated that surgery is either equal to or superior. The use of concurrent chemoradiotherapy, which has become the standard of care in cervical cancer, is another treatment method that warrants additional exploration. It is consequently important to adapt the treatment by a gynecologist based on the features of each individual. It is hoped that the adoption of widespread HPV vaccination programs would result in an even greater reduction in the incidence of this disease in the future. Furthermore, given the fact that most women fall prey to local illness, it is hoped that better techniques such as NACT+ radiation and chemotherapy in cervical cancer with similar survival and without compromising the quality of life can be relevant to this condition.

## Concluding Remarks and Future Directions

In summary, Vaginal cancer in females appears to be a malignancy in their older years of life, specifically the sixth and seventh decades of their lives possess the highest prevalence. It is now clear that approximately 15% of vaginal cancer diagnosed at the age of 50 years, whereas before the age of 40 determine the only 10% vaginal cancer prevalence. However, Squamous cell carcinomas (SCC), as well as adenocarcinomas are two fundamental types of Vaginal Cancer; therefore, Squamous cell carcinomas account for nearly 90% of vaginal cancer cases, while adenocarcinomas account for approximately more than 5%. But most importantly SCC immediately expanded superficially. To detect the etiology of Vaginal cancer, identify the HPV infection, where it enters the host vaginal cells, E6 and E7 genes of the virus play a crucial role in interfering with the tumor suppressor genes (mainly P^53^, and pRb) function. As E6, and E7 protein promoted the carcinogenesis mechanism, and here not only regulate the cellular degradation of P^53^, and pRb but also enhances the cell proliferation along with E6 protein targets the p53 for breakdown and subsequently promote the apoptotic cell death, and DNA repair inhibition, that is indispensable to the continue the lifecycle of the HPV. During the SCC mediated vaginal cancer exhibited the following risk factors notably-first intercourse before the age of seventeen, five or more sexual partners, smoking, prior abnormal cytology, low socioeconomic status, a history of genital warts, and so forth. But to date adenocarcinomas, risk factors are not detected precisely. The most common signs and symptoms of vaginal cancer are bleeding, discharge, and urine retention; in some cases, identify rectal symptoms like constipation or blood in the stool. Pelvic examination is the primary technique to distinctively detect vaginal cancer. If the diagnosis can be done in an early stage of vaginal cancer, then radiation therapy, and surgery to cure this disease. only EBRT, EBRT with brachytherapy, Palliative EBRT combination with chemotherapy are mandatory to address the late stage of this severe disease. At present, several preventive rules are out in diverse scientific reports and added here like HPV vaccination, regular gynecologic examinations, and there are lots of tests are available like Pap test, Cervicography where examine the epithelial lesion of the vagina, and analyzing the vaginal size. More research is required to perfectly meet the molecular mechanism, risk factors, diagnosis, and prevention of Vaginal cancer.

## Author Contributions

Conceptualization by SB, PB, DD, and MI; Writing and main draft preparation by SB, PB, MK, MI, DD, MS, and AM; Figures drawing by TR; Review and editing by TEB, MH, IH, and M-KJ; Visualization and supervision by MR, and BK. The Project Funded by BK. All authors have read and agreed to the published version of the manuscript.

## Funding

This research was supported by Korea Institute of Oriental Medicine (grant number KSN2021240), Basic Science Research Program through the National Research Foundation of Korea (NRF) funded by the Ministry of Education (NRF-2020R1I1A2066868), the National Research Foundation of Korea (NRF) grant funded by the Korea government (MSIT) (No. 2020R1A5A2019413), a grant of the Korea Health Technology R&D Project through the Korea Health Industry Development Institute (KHIDI), funded by the Ministry of Health & Welfare, Republic of Korea (grant number: HF20C0116), and a grant of the Korea Health Technology R&D Project through the Korea Health Industry Development Institute (KHIDI), funded by the Ministry of Health & Welfare, Republic of Korea (grant number: HF20C0038).

## Conflict of Interest

The authors declare that the research was conducted in the absence of any commercial or financial relationships that could be construed as a potential conflict of interest.

## Publisher’s Note

All claims expressed in this article are solely those of the authors and do not necessarily represent those of their affiliated organizations, or those of the publisher, the editors and the reviewers. Any product that may be evaluated in this article, or claim that may be made by its manufacturer, is not guaranteed or endorsed by the publisher.
